# Polyethylene Glycol and Sorbitol-Mediated In Vitro Screening for Drought Stress as an Efficient and Rapid Tool to Reach the Tolerant *Cucumis melo* L. Genotypes

**DOI:** 10.3390/plants12040870

**Published:** 2023-02-15

**Authors:** Maryam Nekoee Mehmandar, Farzad Rasouli, Mousa Torabi Giglou, Seyed Morteza Zahedi, Mohammad Bagher Hassanpouraghdam, Mohammad Ali Aazami, Rana Panahi Tajaragh, Pavel Ryant, Jiri Mlcek

**Affiliations:** 1 Department of Horticulture, Faculty of Agriculture, University of Maragheh, Maragheh 5518779842, Iran; 2 Department of Horticulture, Faculty of Agriculture, University of Mohaghegh Ardabili, Ardabil 5619911367, Iran; 3 Department of Agrochemistry, Soil Science, Microbiology and Plant Nutrition, Faculty of AgriScience, Mendel University in Brno, Zemědělská 1, 613 00 Brno, Czech Republic; 4 Department of Food Analysis and Chemistry, Faculty of Technology, Tomas Bata University in Zlin, Vavreckova 5669, 760 01 Zlin, Czech Republic

**Keywords:** evaluation melon, osmoregulation, simulation, water deficit

## Abstract

An efficient method to instantly assess drought-tolerant plants after germination is using osmoregulation in tissue culture media. In this study, the responses of three Iranian melon genotypes to sorbitol (0.1, 0.2, and 0.4 M) or polyethylene glycol (PEG) (0.009, 0.012, and 0.015 M) were evaluated as drought stress simulators in MS medium. ‘Girke’ (GIR), ‘Ghobadloo’ (GHO), and ‘Toghermezi’ (TOG) were the genotypes. GIR is reputed as a drought-tolerant genotype in Iran. The PEG or sorbitol decreased the coleoptile length, fresh weight, and photosynthetic pigments content while enhancing proline and malondialdehyde (MDA) contents. Protein content and antioxidant enzyme activity were utterly dependent on genotype, osmotic regulators, and their concentration. Coleoptile length, root and shoot fresh weight, root dry weight, proline and MDA content, and guaiacol peroxidase (GPX) activity can be used as indicators for in vitro screening of *Cucumis melo* L. genotypes. The results showed that sorbitol mimics drought stress better than PEG. Overall, our findings suggest that in vitro screening could be an accurate, rapid, and reliable methodology for evaluating and identifying drought-tolerant genotypes.

## 1. Introduction

As desertification increases worldwide, the shortage of water, which detrimentally reduces plant growth, is becoming more drastic [1]. Through the last century, about 70% of drylands (i.e., semi-arid, arid, and hyper-arid lands) have revealed signals of desertification, and between various land-use categories, croplands undergo the highest risk, with ~70% of the area degraded in the worldwide, and 68% of Iran is considered by high and very high desertification potential [2]. Drought stress significantly affects vegetable production, disturbs plant water relationships, reduces leaf size and root growth, and affects plants’ physiological and biochemical features [3]. The crop’s response to water deficit commonly differs among species. It depends on the plant growth phase and other environmental conditions, which are controlled easier in laboratory conditions, and the response can be evaluated better than in the field. In the early stage of growth, drought causes a reduction in plant growth [4]. Furthermore, water deficit significantly diminishes plant growth and development, initiated by the changed water interactions, reduced photosynthesis rate, enhanced reactive oxygen species (ROS) accumulation, their related oxidative destruction, and reductions in cell turgor pressure [5]. As crops are endangered by drought stress for a short or long time, their physiological and molecular strategies are stimulated to protect plant performance under stress [6].

Melon (*Cucumis melo* L.) is an important vegetable cultivated in arid and semi-arid areas of the world [7]. Iran is one of the primary origins of the melon cultivars [8] producing 1,283,699 tons (https://www.fao.org/faostat/en/#search/Canta, loupes and other melons, accessed on 20 September 2022). Many cultivars of melons in the world are generally tolerant to water deficiency; however, water efficiency is a growth-limiting factor in the first growth stage after germination. Native genotypes often have functional genes for adaptation and are tolerant to biological and non-biological stress factors [9,10], which need to be evaluated and identified. Some Iranian genotypes of melon are tolerant to dehydration. The evaluation and identification of tolerant genotypes are essential in developing drought-tolerant melons. A practical method to evaluate plants’ responses to drought stress is in vitro culture, which is a relatively easy mimic of environmental conditions [11]. A common way to reach appropriate tolerant cultivars of crops is by fast screening experiments focusing on quantitative features that can be assessed in vitro within a limited period [12]. 

Polyethylene glycol (PEG) and sorbitol can be used in the in vitro screening for drought conditions [13]. The PEG and sorbitol as osmotic regulators reduce water potential and simulate drought stress in the growing medium. The PEGs are macrogols, a polyether constructed of repeated ethylene glycol units [-(CH2CH2O) n] with grades ranging from 100 to 700 in molecular weight (MW), liquid at 21–24 °C; those between 1000 and 2000 MW are soft solids, and at MW > 2000 are hard crystalline solids with melting points around 63 °C. The high polarity of PEG raises hydrophilicity and increases its water solubility [14]. PEGs do not react with chemicals or biological compounds; they are also non-toxic, cannot be absorbed by plants, and their concentration remains constant throughout the test period [15]. Sorbitol is water-soluble polyhydric alcohol (polyols) having 0.6 sweetness from sucrose and corn syrup and is synthesized from glucose, which is naturally detected in some plants [16].

Studies on the effects of drought in melons have been conducted under field and greenhouse conditions [3,7,17], and no detailed studies have been conducted under in vitro conditions for the screening and selection of *C. melo* genotypes, especially for the assessment of seedlings responses after germination stage. Some studies were conducted on the effects of osmotic materials by creating in vitro and in vivo drought stress in other crops [11,13,15]. Still, no related studies have been traced on the seedlings of melon in the literature so far.

This study aimed to evaluate and simulate drought stress under in vitro culture. Three Iranian melon genotypes were selected with different characteristics against drought stress to compare their in vitro responses with the field conditions. Do the variety that is tolerant to drought in the field have the same reaction in the drought simulated by PEG or sorbitol under in vitro culture? Furthermore, in the current experiment, the response of melon genotypes to sorbitol as polyols was statistically compared to PEG as a polyether compound with different characteristics. On the other hand, this study was arranged to assess the drought-tolerant melons using PEG or sorbitol in vitro by the rapid identification of tolerant genotypes at the early post-germination (seven days) stage after the application of osmolytes, the growing stage which is the most sensitive to drought stress and finally identification of one, or some traits as biomarkers for the screening of drought-tolerant melons under in vitro condition. Our findings supply new insights into the use of in vitro screening in the melon genotypes over drought stress, which would be an efficient and cost-effective method for identifying and selecting many cultivated or wild genotypes to utilize in future breeding programs.

## 2. Results

### 2.1. Morphological Traits

Sorbitol or PEG and genotypes affected coleoptile length, shoot, and root fresh as well as dry weight (Table 1). Coleoptile length was reduced under simulated drought in all three genotypes compared to the control. The longest coleoptile was for TOG control. The maximum reduction belonged to TOG (50%), and the minimum was recorded for GIR (24%) (Table 1).

Shoot FW in all genotypes declined in media with sorbitol and PEG. TOG had the maximum and highest reduction (73%) of shoot FW in 0.015 M PEG; the lowest drop was in the GIR (50%). The control GIR had the minimum shoot FW compared to GHO and TOG controls. Shoot FW was reduced more in all genotypes under the drought simulated by sorbitol than PEG. Root FW was reduced in media with PEG and sorbitol. The TOG produced greater root FW than controls of GHO and GIR. The highest, and most minor reduction of root FW was in TOG (84%) and GIR (65%), respectively. Sorbitol decreased root FW more than PEG (Table 1). 

Shoot DW was affected by increasing PEG and sorbitol concentration; the trait in all media increased compared to the control. The increase was more pronounced in GHO with 0.4 M (7.27%), and TGO with 0.4 and 0.2 M (7.38%, 6.33%) of sorbitol, respectively. GIR had the lowest shoot DW (1.36%). Shoot DW of GHO was 3.15-fold higher than the control and was 1.18-fold higher compared to GIR. Sorbitol increased shoot DW compared to PEG (Table 1).

The root and shoot dry weights increased under drought created with sorbitol and PEG. Root DW of GIR (11.44%) in 0.4 M sorbitol was the highest; the lowest was for TOG (1.52%) controls. The highest root DW was recorded for GIR at 0.4 M sorbitol and was 2.8-fold higher compared to TOG at 0.4 M sorbitol. Generally, sorbitol caused a more significant increase in root DW than PEG treatment (Table 1).

### 2.2. Photosynthetic Pigments

The interaction of sorbitol, or PEG, with genotype affected photosynthetic pigments content (Table 2). There was a decrease in Chl a content when using osmolytes. The highest Chl a content was in control of GHO, TOG, and GIR genotypes with 30.30, 31.72, and 30.58 mg∙kg^−1^ FW, respectively, and in the MS media with 0.015 M PEG; leaves of the GIR genotype had lower Chl a than other genotypes under simulated drought conditions. The reduction of Chl a content was more in GIR (78%) than in TOG (68%) and GHO (54%). Moreover, Chl a content in GIR decreased more quickly than other genotypes in MS media with 0.2 M sorbitol and 0.009 M PEG (Table 2). Chl b decreased in all genotypes exposed to osmolytes. In the MS media with sorbitol and PEG, Chl b content decreased more in GIR than in TOG and GHO. Chl b in GIR decreased 83% in MS media with 0.015 M PEG compared to the control, while the reduction was 70% and 52% for TOG and GHO, respectively. The GIR had more Chl b in controls than other genotypes (Table 2). Chl a and Chl b declined with sorbitol and PEG in the genotypes, and total chlorophyll was reduced more in GIR with PEG and sorbitol treatment than in other genotypes (Table 2). The CARs content was decreased by simulated drought induced with PEG and sorbitol in all genotypes. The most and most minor CARs content were recorded in GHO without osmolytes and GIR with 0.015 M PEG, respectively. The CARs content of GIR was reduced more than GHO and TGO (Table 2).

### 2.3. Proline Content

**Figure 1:**
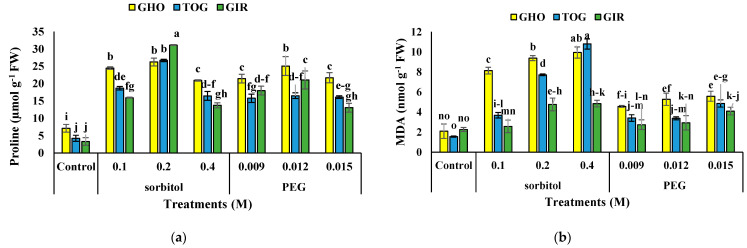

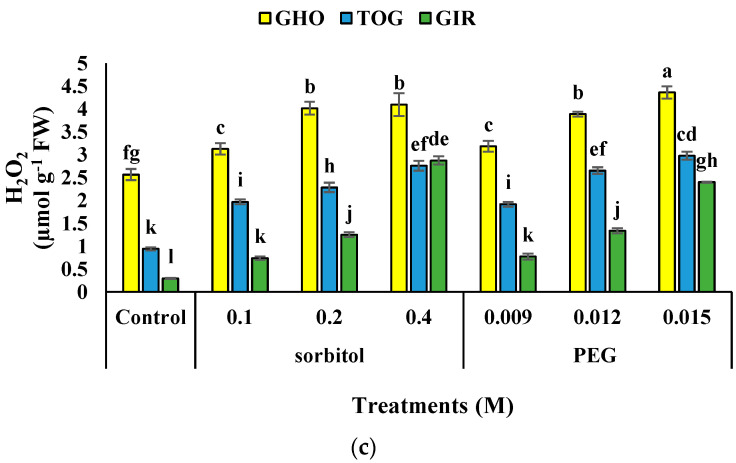
Effects of PEG and sorbitol on proline (**a**), MDA (**b**), and H_2_O_2_ content (**c**) of 3 melon genotypes. Different letters are significantly different based on Duncan’s multiple range test (*p* ≤ 0.05). GHO, TOG, and GIR are the Ghobadloo, Girke, and Toghermezi genotypes, respectively.

The interaction of sorbitol, or PEG, with genotypes, significantly influenced proline content (Table 3). The use of osmolytes led to the enhanced proline content at 0.1, 0.2 M of sorbitol and 0.009 and 0.012 M PEG, decreasing at higher PEG and sorbitol concentrations. However, genotypes under simulated drought produced more proline than controls. The most and the least proline content was in GIR with 0.2 M sorbitol and MS medium without PEG and sorbitol, respectively. In GIR, the highest proline content (8.45-fold) was traced at 0.2 M of sorbitol (Figure 1a).

### 2.4. MDA Content

The interaction of sorbitol or PEG with genotype significantly affected MDA content (Table 3). The MDA in all genotypes increased by applying PEG and sorbitol in the MS medium. The MDA was higher in TGO and GHO at 0.4 M sorbitol, and these genotypes had the most increase of 5.96 and 3.75 times higher amounts compared to controls, respectively. GIR afforded the minor data. MDA content in plants treated with sorbitol was higher compared to plants treated with PEG (Figure 1b).

### 2.5. Hydrogen Peroxide Content

Drought stress significantly increased H_2_O_2_ content in the melon genotypes (Table 3), and the highest H_2_O_2_ content was observed in GHO under 0.015 M PEG, but the lowest was recorded in the control of GIR. GIR had the highest increase in H_2_O_2_ content (870%) under 0.4 M sorbitol, and GHO showed the least related data (69% increase) supplemented with 0.015 M PEG compared to the control (Figure 1c).

### 2.6. TAA

**Figure 2:**
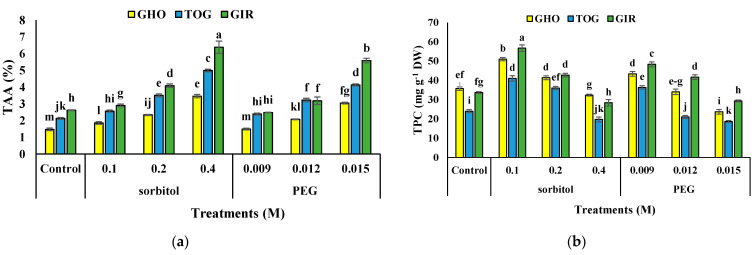

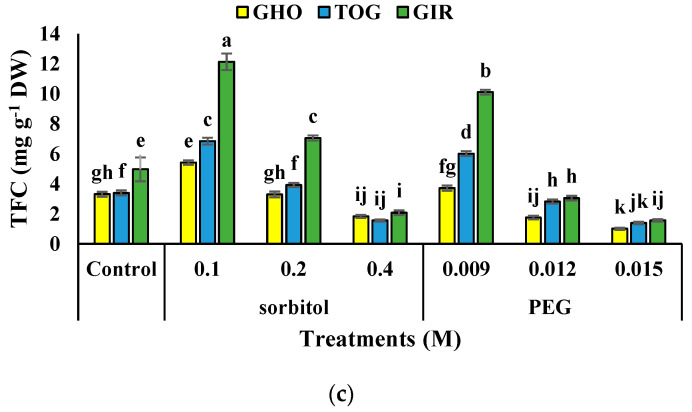
Effects of PEG and sorbitol on TAA (**a**), TPC (**b**), and TFC (**c**) of 3 melon genotypes. Different letters are significantly different based on Duncan’s multiple range test (*p* ≤ 0.05). GHO, TOG, and GIR are the Ghobadloo, Girke, and Toghermezi genotypes, respectively.

TAA was significantly affected by drought stress (Table 3). In all three genotypes, an enhancement was observed in TAA content by increasing osmotic materials over to the control. The highest TAA content was obtained in the highest sorbitol concentration of the GIR genotype and the lowest was for GHO control × 0.009 M PEG. The most significant increase in TAA content was obtained in GIR (144%) under the highest concentration of sorbitol (Figure 2a). 

### 2.7. TPC and TFC

TPC and TFC content in all the genotypes were added under moderate drought stress. The utmost TPC and TFC content was detected in GIR subjected to 0.1 M of sorbitol, while the least level in both traits was detected in TOG and GHO exposed to 0.015 M of PEG, respectively. The uppermost increase in TPC was traced in the GHO genotype (115%), and TFC was noted in GIR (677%). The lowest related data was recorded in GIR for TPC (93%) and TFC (391%) at 0.1 M of sorbitol treatment (Figure 2b,c).

### 2.8. TSP Content

**Figure 3:**
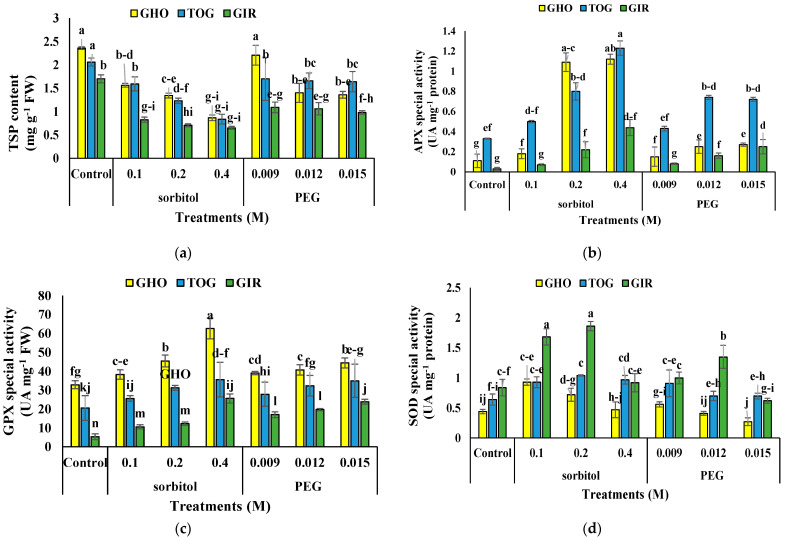
The effects of PEG and sorbitol on total protein content (**a**), APX activity (**b**), GPX activity (**c**), and SOD activity (**d**) of three melon genotypes. Different letters are significantly different based on Duncan’s multiple range test (*p* ≤ 0.05). GHO, TOG, and GIR are the Ghobadloo, Girke, and Toghermezi genotypes, respectively.

The interaction of sorbitol or PEG with genotype affected TSP content (Table 4). TSP decreased in MS media containing sorbitol and PEG compared to the control, but there was no difference at 0.009 M PEG in GHO. The highest TSP was in the GHO control, supplemented with 0.009 M PEG, and in the TOG control. GHO had the most reduction, up to 63.55%, and TOG had the least reduction, up to 59.70%, at 0.4 M of sorbitol, compared to the control. Sorbitol decreased total protein more than PEG (Figure 3a).

### 2.9. Antioxidant Enzyme Activity

Sorbitol or PEG affected antioxidant enzyme activity individually and in interaction with the genotype (Table 4). Both sorbitol and PEG stimulated APX activity, and the activity induced by sorbitol was much more significant than PEG. The highest APX activity was in TOG under 0.4 M sorbitol and GHO under 0.2 and 0.4 M sorbitol; the most APX activity was in GIR (13.6 times) (Figure 3b).

The GPX activity increased in the MS medium with PEG and sorbitol in all concentrations and genotypes. The highest GPX activity was in GHO in MS medium with 0.4 M sorbitol, and the highest increase was in GIR with 0.4 M sorbitol, nearly 3-times more than GHO and TOG (Figure 3c).

The SOD activity was increased by adding PEG and sorbitol to the MS medium in all genotypes; however, there was no significant difference with the higher concentration of treatments. The most SOD activity was in GIR in MS medium with 0.2 and 0.1 M sorbitol. In contrast, the highest was in GHO with 0.1 M sorbitol. The sorbitol-induced activity of SOD was more than PEG (Figure 3d).

### 2.10. Correlations, Principal Component Analysis, and Loading Biplot Analysis

**Figure 4:**
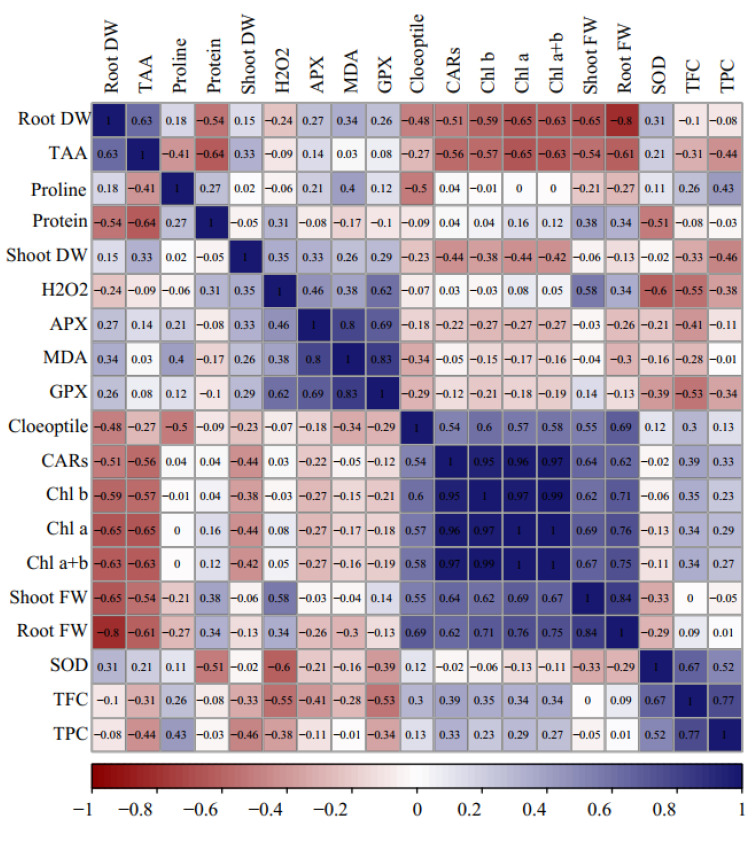
Heat map of Pearson’s correlation analysis for the response of Cucumis melo genotypes to the sorbitol and PEG-induced drought stress under in vitro conditions. Heat map representing coleoptile length, shoot and root FW and DW, chlorophyll a (Chl a), chlorophyll b (Chl b), total chlorophyll (Total Chl), carotenoids (CARs), proline content, malondialdehyde (MDA), H_2_O_2_ content, total soluble protein content, guaiacol peroxidase (GPX) activity, ascorbate peroxidase (APX) activity, superoxide dismutase (SOD) activity, total flavonoids content (TFC), and total phenol content (TPC).

The heat map of Pearson correlations analysis explained significant positive and negative correlations among the evaluated traits at 5% and 1% of *p*-values. Significant positive correlations were recorded among coleoptile length, shoot FW, root FW, and photosynthetic pigments, and negative correlations with shoot DW, root DW, proline, MDA, total protein, APX, and GPX activity. Moreover, root DW was positively correlated with TAA but negatively correlated with root FW. Similarly, GP, APX, H_2_O_2_, and MDA represented significant positive correlations with each other (Figure 4).

**Figure 5:**
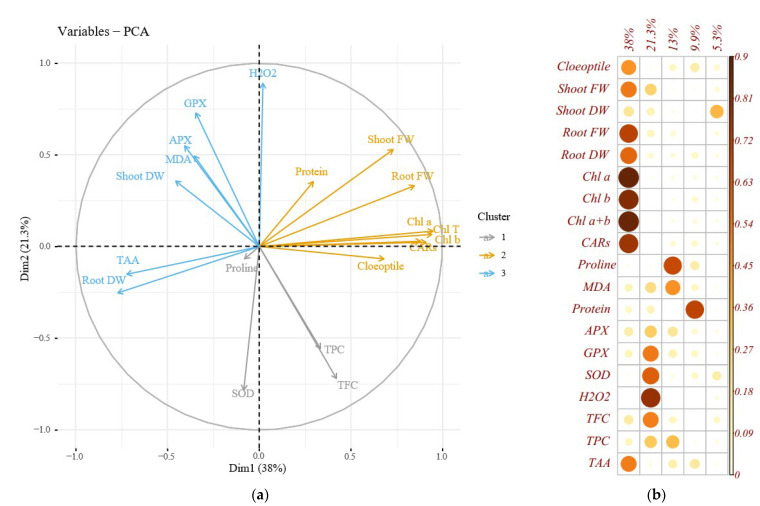
Loading biplot of the evaluated traits (**a**) and Principal component analysis (**b**) of the enzymatic antioxidants pool, the morphological and biochemical changes in Cucumis melo genotypes under sorbitol and PEG-induced drought stress in vitro condition. Heat map representing coleoptile length, shoot, and root FW and DW, chlorophyll a (Chl a), chlorophyll b (Chl b), total chlorophyll (Total Chl), carotenoids (CARs), proline content, malondialdehyde (MDA), H_2_O_2_ content, total soluble protein content, guaiacol peroxidase (GPX) activity, ascorbate peroxidase (APX) activity, superoxide dismutase (SOD) activity, total flavonoids content (TFC), and total phenol content (TPC).

The loading plot of the studied variables revealed that the correlated variables were placed on the plot at close distances (Figure 5). The loading biplot revealed three clusters among the evaluated traits. Cluster 1 included root FW, TAA, shoot FW, MDA, APX, GPX, and H_2_O_2_; cluster 2 consisted of total soluble protein, shoot FW, root FW, coleoptile length, and photosynthesis pigments, and cluster 3 contained TPC, TFC, SOD, and proline content (Figure 5a). 

The principal component analysis (PCA) was conducted to reveal the pattern of variation among the assessed morphological and physiological traits and to list a more effective interpretation of the weight of each attribute in the total variation. In the present experiment, 4 PCs elucidated 82.2% of the total variance. The PC1 was the principal and most efficient component, responsible for nearly 38% of the exploited total variance. The most influential variables in the PC1 were Chl a, Chl b, total Chl, CARs, root DW, root FW, shoot FW, coleoptile length, and TAA. Nevertheless, PC2 accounted for 21.3% of variations in which H_2_O_2_, SOD, and GPS were most effective. PC3 described 13% of the total variance, and proline content was a prominent trait. Total soluble protein content was the primary trait in PC4 representing 17.22% of the total variance (Figure 5b).

## 3. Discussion

Coleoptile length, shoot, and root FW were adversely affected by adding PEG and sorbitol to the MS medium. However, shoot and root DW were positively affected. Sorbitol, or PEG, decreased water potential and simulated drought, adversely affecting cell division, cytoplasm volume, turgor pressure, and nutritional balance. Intensifying the drought can reduce cytokinins, increase ABA levels that influence cell division, reduce fresh weight, and restrict plant growth [18,19]. Under water stress conditions, stem and root meristematic cells are affected, cell division and elongation are immediately interrupted, and stem and root length are reduced. Furthermore, the turgor pressure diminishes under water stress necessary for cell extension [20]. A decreased dry mass is probably due to dehydration, and new osmolyte synthesis may be required to protect higher osmotic levels and continue water uptake [17].

In general, photosynthetic pigment contents were reduced in genotypes under water deficiency. Pigment reduction during drought can increase abscisic acid, ethylene, chlorophyllase, and peroxidase activities [21]. One of the reasons for the chlorophyll lessening owing to water deficit is that it causes lipid peroxidation by producing ROS such as peroxidase and hydrogen peroxidase [22]. A decline in chlorophyll content was reported in melon [3,7,17] and cucumber [23]. GIR had the most significant decrease in photosynthetic pigment content in most treatments, which could be related to leaves being pale green during the dry season.

Proline plays a dominant role in maintaining plant osmotic potential and cell osmotic adjustment [24]; it has a role in plant tolerance under biotic and abiotic challenges via fortifying the antioxidant activity and ROS scavenging potential [7]. Proline also has a vital role in stabilizing the three-dimensional structure of proteins, inhibiting the peroxidation of membrane lipids, and regulating cytosolic acidity [25]. Another reason for proline increment is ROS as a part of ABA signaling, and on the other hand, ABA enhances under the water deficit stress in plants [26]. Proline content increased with higher PEG or sorbitol treatments in the genotypes. As known, the accumulation of proline depended on genotype and osmotic substances` interaction, and more proline in GIR indicated greater tolerance than others under simulated drought.

Antioxidants protect cells against free radicals and have therefore been considered in numerous studies as a way to improve plant defense responses [27]. The TAA content of the three genotypes under drought stress was simulated using sorbitol and PEG. The results showed that drought stress increased TAA, and the sorbitol increase rate was higher than PEG. TAA enhancement is reported to be caused by activating the signal transduction pathways, promoting the antioxidant enzyme related genes’ expression, and improving their activity [28]. Our findings are further supported by previous studies that reported higher TAA in plants under drought stress. The current results showed that TAA as ROS scavengers increase significantly in plants under drought stress treatment, which can be part of the adaptive strategy of melon genotypes under a stress environment, which is consistent with the results of Shirani Bidabadi and Sharifi [29].

The accumulation of antioxidant compounds such as flavonoids and other phenolics is one of the general responses to abiotic stresses in plants [30]. Total phenolic content is the primary determinant of antioxidant potential [29]. It was reported that phenylalanine ammonia-lyase activity increased under abiotic stress, which altered the pathway of secondary metabolites to the production of phenolic compounds [28]. Flavonoids are also considered defense metabolites against environmental stresses and efficient secondary ROS scavenging systems [30]. The present results showed that the use of osmolytes in low concentrations increased TPC and TFC, but both decreased in a higher concentration of sorbitol and PEG. According to Josipović et al. [31], the content of polyphenols in plants depends on biological factors such as genotype and plant organ as well as the edaphic and environmental conditions such as temperature, salinity, water availability, and light intensity, which is in agreement with our results.

The increased MDA generation due to membrane lipid peroxidation indicates the damage caused by oxidative stresses [7]. Drought stress triggers the peroxidation of thylakoid’s anchored glycolipids leading to the over-production of diacylglycerol, triacylglycerol, and free fatty acids and hence the increased MDA production in plant tissue [32]. Plants with higher drought tolerance produce less MDA because of their ability to maintain membrane integrity [7,33]. GIR was likely more tolerant than the others due to less MDA content under drought.

Water shortage caused oxidative stress in melon genotypes, which led to high H_2_O_2_ accumulation. Disruption of the electron transport chain in the chloroplast, mitochondria, and plasma membrane under drought stress may lead to increased ROS formation [34]. H_2_O_2_ is a toxic compound for cells and must be quickly converted into water and oxygen by the antioxidant defense system. Otherwise, it can damage the cell membrane, protein structure, and DNA through lipid peroxidation and even prevent photosynthesis and enzyme activity [7]. In addition to damaging plant cells, free radicals act as signaling molecules and activate cell defense responses against stresses [35]. In our research, drought stress caused oxidative damage and increased H_2_O_2_ levels in the melon genotypes. A similar result was observed in yarrow species [27], strawberries [36], and wheat [37].

The intensified simulated drought conditions reduced the total protein content of plants [17]. The reduction in total soluble protein content with the abiotic stress can be assigned to the high accumulation of ROS, which ultimately causes the oxidation of proteins and a quick reduction in the biosynthesis and/or functioning of some structural proteins [38]. The total protein content of plants under PEG and sorbitol treatments decreased compared to the control. Total protein content decreased in genotypes under MS medium with sorbitol more than PEG, probably due to greater penetration of sorbitol at high concentrations.

The antioxidant enzymes APX, GPX, and SOD special activity increased with water deficiency. Sorbitol enhanced enzyme activities more than PEG, and genotype responses differed from control, so GIR had the most APX, GPX, and SOD activities. Increasing APX, GPX, and SOD special activity under simulated drought in response to ROS accumulation was probably due to the increased expression of antioxidant enzyme related genes and the more action of enzymes by activating the transcriptional signaling pathways [39]. Since hydrogen peroxide molecules produced under oxidative stress are highly toxic, the antioxidant enzymes have essential roles in neutralizing the harmful effect of hydrogen peroxide [28].

## 4. Materials and Methods

Seeds of the C. melo Iranian genotypes: Ghobadloo (GHO), Girke (GIR), and Toghermezi (TOG) were collected from Ajbashir (37.4788° N, 45.8929° E), Boukan (36.5187° N, 46.2094° E), and Isfahan (32.6539° N, 51.6660° E), respectively. For GIR, dryland farming is the norm; for TOG and GHO, a light to moderate irrigation regime is commonly used. All seeds were disinfected with 10% sodium hypochlorite (NaOCl) solution for 15 min and subsequently washed with distilled water and air-dried. Seeds were cultured in ¼ MS medium (Murashige and Skoog 1962). After germination and production of the true leaves, seedlings were transferred to MS media containing sorbitol (Merck, Darmstadt, Germany) and PEG 6000 (Merck, Darmstadt, Germany), and each seedling was transferred in a vessel with 50 mL of the media and then placed in a growth chamber. The growth chamber had a photoperiod of 16/8 h (light/dark), a temperature of 25 °C, and relative air humidity of 70 ± 5%. The sorbitol was added into the media at the concentrations of 0.1, 0.2, and 0.4 M, determining the following values of medium osmotic potentials −0.88, −1.12, and −1.80 MPa, respectively, estimated by the Reid [40] formula. The PEG was also added into the media at three different concentrations of 0.009, 0.012, and 0.015 M, corresponding to the medium osmotic potentials of −0.55, −0.87, and −1.2 MPa, as determined by Michel and Kaufmann [41] formula. They were added to the MS medium, containing 8 g L^−1^ agar and 30 g L^−1^ sucrose. The control was MS medium without sorbitol and PEG. Sampling was performed 7 days after transferring the seedling into the vessel. In other words, the seedling was placed under the PEG and mannitol treatments for seven days.

### 4.1. Morphological Traits

The coleoptile length and shoot and root fresh weights were determined. Tissues were dried in a convection oven at 70 °C for 24 h for dry weight determination.

### 4.2. Photosynthesis Pigments

Chlorophylls (Chl a and b) and carotenoids (CARs) contents were determined spectrophotometrically (UV-1800 Shimadzu, Kyoto, Japan) using equations described by Arnon [42]. Leaf samples (0.5 g) were ground in liquid nitrogen, suspended in 10 mL of 80% acetone, mixed and extracted in 2 mL of extract, and poured into tubes. The concentration of these photosynthesis pigments was determined by measuring the extinction of the extract at the major red absorption maxima of 664 nm, 647 nm, and 470 nm, and the photosynthesis pigments content was estimated with the following equations:

Chlorophyll a (mg g^−1^ FW) = [12.7(A663) − 2.69(A645)]

Chlorophyll b (mg kg^−1^ FW) = [21.50(A645) − 5.10(A663)]

Carotenoids (mg kg^−1^ FW) = [1000(A470) − 1.82Chl a − 85.02Chl b]/198

A663: absorbance at 663 nm, A645 at 645 nm, A470 at 470 nm. The absorbance coefficients are 12.7 and 5.10 for the red peak of Chl a, and 2.69 and 21.50 are the absorbance coefficients for the red peak of Chl b; 1000, 1.82, and 85.02 are the absorbance coefficients for the blue peak of carotenoids.

### 4.3. Proline Content

Fresh leaves (0.5 g) were ground with liquid nitrogen. Then, 10 mL of 3% sulphosalicylic acid was added and centrifuged at 1120× *g* for 20 min at 4 °C, and 2 mL of ninhydrin acid and 2 mL of glacial acetic acid were added to the supernatant. The mixture was placed in a boiling water bath for 60 min, then removed and immediately cooled in ice bath for 5 min. Then, 4 mL of toluene was added, and the mixture was vortexed for 20 s. Absorbance was recorded at 520 nm [43]. Proline concentration was determined using a proline calibration curve and expressed as µmol g^−1^ FW.

### 4.4. Malondialdehyde Content (MDA)

The amount of malondialdehyde (MDA) was estimated following Heath and Packer [44]. A sample of 0.5 g of fresh leaves was extracted in 1.5 mL trichloroacetic acid 1% *w*/*v* (TCA) and centrifuged at 1120× *g* for 10 min, and 1 mL thiobarbituric acid 0.1% *w*/*v* (TBA) was added to 0.5 mL of the supernatant. The mixture was boiled at 95°C for 30 min and cooled on ice for 15 min. Absorbance was recorded at 532 and 600 nm. MDA content was calculated as in the below equation:

$$\mathrm{MDA}=\left[\frac{\left(\text{A}532-\text{A}600\right)}{155}\right]\times 1000$$

A532: absorbance at 532 nm, A600: absorbance at 600 nm

### 4.5. H_2_O_2_ Content

A sample of 0.5 g of leaf fresh samples was homogenized with 5 mL trichloroacetic acid (0.1% *w*/*v*) on an ice bath. The homogenate was centrifuged at 16,099× *g* for 20 min. Then, 500 μL of the supernatant was mixed with 500 μL potassium phosphate buffer (10 mM, pH = 6.8) and 1000 μL KI (1M). Later, the absorbance was read spectrophotometrically at 390 nm. H_2_O_2_ contents were measured through a standard calibration curve previously made from several H_2_O_2_ concentrations and calculated as μM g^−1^ FW [45].

### 4.6. Total Antioxidant Activity (TAA)

TAA was determined using the 1,1-diphenyl-2-picrylhydrazyl (DPPH) method. A sample of 1 g of the fresh tissue samples was extracted with 2 mL of 80% methanol and centrifuged at 21,913× *g* for 15 min. Then, 100 µL of the extract was mixed with 180 µL of DPPH (0.1 M), placed in dark conditions for 30 min, and read at 517 nm by a UV spectrophotometer, and TAA was calculated using the following equation [46]:

$$\%\mathrm{TAA}=\frac{\mathrm{OD}\;\mathrm{control}-\mathrm{OD}\;\mathrm{sample}}{\mathrm{OD}\;\mathrm{control}}\times 100$$

OD DPPH + OD methanol = OD control

OD: the absorbance by a UV spectrophotometer 

### 4.7. Total Phenolics Content (TPC)

A sample of 1 g of fresh leaf tissue was extracted by acidic methanol and centrifuged at 16,099× *g* for 10 min. Then, 1.59 mL of distilled water, 100 μL of 10% Folin–Ciocalteu and 20 μL of the extract were mixed and stored for 10 min. Then, 300 μL of 7.5% sodium carbonate was added to the mixture and kept in the dark for two hours. Ultimately, the absorbance was determined at 765 nm with a UV spectrophotometer, and TPC was calculated as mg g^−1^ FW using a standard calibration curve via several galic acid concentrations [47]. 

### 4.8. Total Flavonoids Content (TFC)

A sample of 1 g of fresh tissue was homogenized in methanol 80% and centrifuged at 21,913× *g* for 15 min. Then, 200 μL of the supernatant, 600 μL of 95% methanol, 40 μL of 10% aluminum chloride, 40 μL of 1 M potassium acetate, and 1120 μL of distilled water were mixed and kept at room temperature for 40 min. The absorbance was recorded at 415 nm, and TFC presented as mg g^−1^ FW utilizing a standard calibration curve made from several quercetin concentrations [48]. 

### 4.9. Total Soluble Protein (TSP) Content

TSP was determined with fresh leaf samples (0.2 g), which were ground in liquid nitrogen and homogenized in 1.5 mL of 50 mM Na buffer phosphate (pH 7.8), including 1 mM EDTA and 2% (*w*/*v*) polyvinylpolypyrrolidone. The homogenate was centrifuged at 1344× *g* for 15 min at 4 °C. Supernatants were used for total soluble protein and extracted for enzyme activity. Total protein content was measured following Bradford [49], with bovine serum albumin (BSA) as a standard with the serial solutions containing 0, 0.2, 0.4, 0.6, 0.8, or 1 mg·mL^−1^ to which 100 μL of Bradford solution was added. The Bradford reagent was prepared with 50 mg of coomassie brilliant blue G-250 dissolved in 50 mL of methanol, and 100 mL of 85% (*w*/*v*) phosphoric acid. The solution was added to 850 mL H_2_O and filtered through Whatman filter paper #1. Finally, 1000 μL of the Bradford reagent was added to 50 μL protein buffer phosphate samples, mixed, and incubated for 5 min. Absorbance was read (A595 nm) with a spectrophotometer (model 100, Cary, Richmond, VA, USA). 

### 4.10. Antioxidant Enzymes Activity

Antioxidant enzymes were quantified with ascorbate peroxidase activity (APX) assayed according to Nakano and Asada [50] with the oxidation of ascorbate by APX. The reaction mixture included 2550 μL 0.5 mM ascorbate, 450 μL 3% H_2_O_2_, and 50 μL extract and was recorded at 290 nm. The APX activity was defined as µM min^−1^ mg^−1^ FW or UA mg^−1^ FW.

Guaiacol peroxidase activity (GPX) was measured by reducing H_2_O_2_ with oxidation of guaiacol following Chance and Maehly (1955). The reaction mixture was 1500 μL sodium buffer phosphate (100 mM) pH:7, 120 μL H_2_O_2_ (15 mM), 480 μL guaiacol (20 mM), and 50 μL of enzyme extract. The absorbance raise was recorded at 470 nm. The GPX activity was defined as µM min^−1^ mg^−1^ FW or UA mg^−1^ FW.

Superoxide dismutase activity (SOD) was determined according to Giannopolites and Rice (1977). The mixture contained 100 μL sodium carbonate (1.5 mM), 200 μL methionine (0.2 M), 100 μL EDTA (3 mM), 1500 μL sodium phosphate buffer (0.1 M), 900 μL distilled water, 100 μL nitro blue tetrazolium (2.25 mM), and 50 μL extract enzyme. The reaction started with adding 100 μL riboflavin (60 μM). The mixture was incubated under light for 15 min, then the absorbance was recorded at 560 nm. The reaction mixture having no enzyme extracts was the control. The GPX activity was defined as µM min^−1^ mg^−1^ FW or UA mg^−1^ FW.

### 4.11. Statistical Experiments

The experiment was arranged as a factorial based on a completely randomized design with four replications. The data were subjected to ANOVA in a general linear method (GLM) using SAS software (ver. 9, SAS Institute, Cary, NC, USA). Moreover, R software did Pearson correlation and cluster dendrogram heat maps (version 4.1.2), (URL https://cran.um.ac.ir/, accessed on 10 December 2021. R packages of ‘corrplot’ (Visualization of a Correlation Matrix, version 0.91; https://github.com/taiyun/corrplot, accessed on 10 December 2021) and ‘gplots’ (Various R Programming Tools for Plotting Data, version 3.1.1; https://github.com/talgalili/gplots/issues, accessed on 10 December 2021)).

## 5. Conclusions

The evaluated growth and biochemical characters were affected by PEG and sorbitol-induced drought stress. Based on the current results (especially PCA and correlation analysis), coleoptile’s length, root and shoot FW, root DW, proline, photosynthesis pigments, TAA, proline content, H_2_O_2_, SOD, GPX activity, and total soluble protein can be used as biomarkers for in vitro drought screening of *C. melo* genotypes. In contrast, protein content, photosynthesis pigment content, and SOD activity are less reliable for in vitro selection because the characteristics rely on genotypes, and the response of the assessed genotypes was different from drought stress simulated by PEG and sorbitol. Based on the response of genotypes to the drought regulators, GIR was more tolerant than GHO and TOG. GIR is reputed as a drought-tolerant genotype in Iran. Furthermore, the biomarker mentioned above showed that GIR was more drought-tolerant than GHO and TOG. This could be another reason these traits can be used as morphological, biochemical, and physiological markers to identify drought-tolerant melons under in vitro culture screening methodologies. These findings showed that sorbitol mimicked drought better than PEG. Although sorbitol gradually penetrates cells and may have caused toxicity in higher concentrations, the penetration can be minimized by the reduced exposure like in this experiment. The PEG may affect root development because the compound is sticky and stiff. Our findings revealed that in vitro screening can be considered a reliable and rapid selection methodology for the numerous melon genotypes in the laboratory. 

## Figures and Tables

**Table 1:** Effect of sorbitol and PEG-induced drought stress on morphological traits of three melon genotypes under in vitro culture conditions.

Genotype	Treatment	Concentration (M)	Character				
			Coleoptile Length (mm)	Shoot FW (g Plantlet^−1^)	Shoot DW (%)	Root FW (g Plantlet^−1^)	Root DW (%)
GHO	Control	0	39.82 ± 4.18 ^b^	0.913 ± 0.04 ^c^	1.360 ± 0.38 ^e^	0.293 ± 0.03 ^b^	4.131 ± 1.11 ^i–k^
	Sorbitol	0.1	30.24 ± 0.87 ^f–i^	0.596 ± 0.03 ^d^	1.850 ± 0.33 ^d,e^	0.213 ± 0.02 ^c,d^	5.113 ± 0.28 ^g–k^
		0.2	25.88 ± 0.90 ^i–l^	0.516 ± 0.11 ^d,e^	5.103 ± 2.27 ^b,c^	0.136 ± 0.04 ^e–g^	8.387 ± 1.72 ^b–e^
		0.4	25.57 ± 0.77 ^j–l^	0.420 ± 0.02 ^f,g^	7.270 ± 1.74 ^a^	0.09 ± 0.02 ^f–h^	9.037 ± 1.41 ^b^
	PEG	0.009	31.65 ± 2.12 ^d–g^	0.59 ± 0.11 ^d^	1.757 ± 0.44 ^e^	0.206 ± 0.01 ^c,d^	4.723 ± 0.66 ^h–k^
		0.012	29.18 ± 1.37 ^f–j^	0.396 ± 0.06 ^f,g^	2.330 ± 0.32 ^d,e^	0.213 ± 0.01 ^c,d^	6.473 ± 0.39 ^e–h^
		0.015	24.23 ± 0.68 ^k,l^	0.37 ± 0.04 ^g,h^	2.873 ± 0.35 ^d,e^	0.11 ± 0.06 ^d,e^	6.547 ± 0.41 ^e–h^
TOG	Control	0	44.50 ± 4.30 ^a^	1.173 ± 0.09 ^a^	2.583 ± 0.39 ^c–e^	0.446 ± 0.07 ^a^	1.523 ± 0.13 ^l^
	Sorbitol	0.1	35.64 ± 2.41 ^c,d^	1.053 ± 0.01 ^b^	3.613 ± 1.69 ^c,d^	0.233 ± 0.01 ^c^	3.843 ± 1.36 ^k^
		0.2	34.95 ± 1.90 ^c–e^	0.500 ± 0.01 ^d–f^	6.330 ± 0.49 ^a,b^	0.200 ± 0.02 ^c,d^	5.273 ± 0.22 ^g–k^
		0.4	31.31 ± 2.16 ^d–g^	0.400 ± 0.05 ^f,g^	7.383 ± 0.86 ^a^	0.070 ± 0.04 ^h^	8.917 ± 0.88 ^b,c^
	PEG	0.009	27.24 ± 0.72 ^g–k^	0.413 ± 0.01 ^e,f^	2.680 ± 0.08 ^d,e^	0.126 ± 0.01 ^e–h^	6.257 ± 0.78 ^f–i^
		0.012	26.59 ± 1.84 ^h–k^	0.316 ± 0.14 ^g–i^	3.563 ± 0.89^c,d^	0.120 ± 0.01 ^e–h^	6.91 ± 0.07 ^f–i^
		0.015	22.23 ± 0.81 ^l^	0.320 ± 0.03 ^g–i^	5.657 ± 0.88 ^b^	0.096 ± 0.01 ^f–h^	7.060 ± 0.99 ^c–g^
GIR	Control	0	37.12 ± 2.45 ^b,c^	0.263 ± 0.05 ^h,i^	1.630 ± 0.29 ^e^	0.206 ± 0.02 ^c,d^	4.263 ± 0.27 ^j,k^
	Sorbitol	0.1	32.96 ± 0.20 ^c–f^	0.263 ± 0.04 ^h,i^	1.840 ± 0.37 ^d,e^	0.140 ± 0.01 ^e,f^	8.620 ± 1.19 ^b–d^
		0.2	31.52 ± 0.84 ^d–g^	0.243 ± 0.01 ^i–k^	2.297 ± 0.91 ^c–e^	0.110 ± 0.01^e–h^	9.083 ± 0.60 ^b^
		0.4	29.43 ± 2.00 ^f–j^	0.183 ± 0.01 ^j,k^	5.637 ± 0.02 ^b,c^	0.073 ± 0.01 ^h^	11.44 ± 1.35 ^a^
	PEG	0.009	34.99 ± 2.01 ^c–e^	0.233 ± 0.02 ^i,j^	2.150 ± 0.16 ^d,e^	0.163 ± 0.02 ^d,e^	5.863 ± 0.23 ^f–j^
		0.012	30.60 ± 0.44 ^e–h^	0.170 ± 0.04 ^j,k^	2.397 ± 0.15 ^c–e^	0.133 ± 0.03 ^e–g^	6.803 ± 0.48 ^d–g^
		0.015	28.19 ± 0.82 ^g–k^	0.133 ± 0.01 ^k^	3.553 ± 0.13 ^c,d^	0.090 ± 0.01 ^f–h^	7.567 ± 0.53 ^b–f^
S.O.V.							
Genotype			42.210 **	0.901 **	17.612 **	0.022 **	12.828 **
Drought			199.411 **	0.310 **	17.694 **	0.046 **	38.264 **
Genotype × Drought			38.582 **	0.095 **	6.582 **	0.011 **	8.639 **
Error			5.664	0.004	1.107	0.001	1.108
C.V. (%)			7.64	14.56	29.91	19.19	15.57

** indicated significance at 1% probability levels. Mean with the same letter are not significantly different by Duncan grouping at (*p* ≤ 0.05) in each column. Ghobadloo (GHO), Girke (GIR), and Toghermezi (TOG). S.O.V. and C.V. refer to the source of variation and coefficient of variation, respectively.

**Table 2:** The effect of drought stress induced by sorbitol and PEG on photosynthesis pigments content in three melon genotypes under in vitro culture conditions.

Genotype	Treatment	Concentration (M)	Character			
			Chl a (mg kg^−1^ FW)	Chl b (mg kg^−1^ FW)	Chl a + b (mg kg^−1^ FW)	CARs (mg kg^−1^ FW)
GHO	Control	0	30.60 ± 0.44 ^a^	12.61 ± 0.07 ^b,c^	43.22 ± 0.51 ^a^	7.543 ± 0.66 ^a^
	Sorbitol	0.1	23.36 ± 1.90 ^c^	9.743 ± 0.55 ^e^	33.10 ± 2.44 ^c^	5.423 ± 0.27 ^c^
		0.2	18.12 ± 1.05 ^d^	6.650 ± 0.44 ^f^	24.77 ± 1.48 ^d^	4.407 ± 0.32 ^d,e^
		0.4	14.24 ± 2.03 ^e,f^	5.913 ± 0.74 ^f–h^	20.16 ± 2.78 ^e,f^	3.723 ± 0.60 ^e,f^
	PEG	0.009	18.27 ± 1.04 ^d^	6.433 ± 0.06 ^f,g^	24.70 ± 0.99 ^d^	4.640 ± 0.37 ^c,d^
		0.012	16.40 ± 0.29 ^d,e^	6.400 ± 1.37 ^f,g^	22.80 ± 1.56 ^d,e^	3.343 ± 0.05 ^f,g^
		0.015	13.92 ± 0.19 ^f,g^	5.953 ± 0.08 ^f–h^	19.87 ± 0.27 ^e–g^	2.960 ± 0.12 ^f,g^
OG	Control	0	31.72 ± 1.66 ^a^	13.38 ± 0.74 ^b^	45.10 ± 2.39 ^a^	6.907 ± 0.71 ^a,b^
	Sorbitol	0.1	27.33 ± 0.10 ^b^	11.20 ± 0.04 ^d^	38.54 ± 0.14 ^b^	6.463 ± 0.84 ^b^
		0.2	12.52 ± 1.13 ^f–h^	5.343 ± 1.76 ^f–i^	17.86 ± 2.08 ^f–h^	2.970 ± 0.29 ^f,g^
		0.4	9.21 ± 0.65 ^i,j^	4.937 ± 0.43 ^g–j^	13.27 ± 2.03 ^j–l^	2.687 ± 0.53 ^g–i^
	EG	0.009	11.87 ± 0.87 ^f–i^	5.247 ± 0.42 ^f–i^	17.11 ± 1.26 ^f–i^	2.770 ± 0.24 ^g,h^
		0.012	11.63 ± 1.28 ^g–i^	4.737 ± 0.47 ^h–j^	16.36 ± 1.75 ^g–j^	2.643 ± 0.29 ^g–i^
		0.015	10.09 ± 1.27 ^h,i^	4.017 ± 0.62 ^i–k^	14.11 ± 1.53 ^i–l^	2.627 ± 0.39 ^g–i^
GIR	Control	0	30.58 ± 1.91 ^a^	15.32 ± 0.81 ^a^	45.89 ± 2.67 ^a^	7.200 ± 0.33 ^a,b^
	Sorbitol	0.1	26.73 ± 1.26 ^b^	11.70 ± 0.60 ^c,d^	38.43 ± 1.85 ^b^	6.670 ± 0.37 ^a,b^
		0.2	9.563 ± 1.50 ^i,j^	4.263 ± 0.40 ^i–k^	13.59 ± 0.81 ^i–l^	2.750 ± 0.18 ^g,h^
		0.4	9.327 ± 0.53 ^i,j^	3.707 ± 0.53 ^j–l^	11.46 ± 0.36 ^k–m^	2.583 ± 0.33 ^g–i^
	PEG	0.009	11.17 ± 0.13 ^h,i^	4.753 ± 0.22 ^h–j^	15.93 ± 0.09 ^h–j^	2.553 ± 0.09 ^g–i^
		0.012	7.300 ± 0.52 ^j,k^	3.010 ± 0.28 ^j–l^	10.31 ± 0.79 ^l,m^	1.823 ± 0.31 ^h,i^
		0.015	6.643 ± 1.00 ^k^	2.650 ± 0.28 ^l^	9.293 ± 1.28 ^m^	1.747 ± 0.23 ^i^
S.O.V.						
Genotype			126.906 **	7.460 **	195.443 **	5.202 **
Drought			624.092 **	125.034 **	1305.071 **	31.152 **
Genotype × Drought			23.462 **	4.756 **	44.513 **	1.588 **
Error			1.983	0.658	3.885	0.251
C.V. (%)			8.50	11.51	8.35	12.46

** indicated significance at 1% probability levels. Mean with the same letter are not significantly different by Duncan grouping at (*p* ≤ 0.05) in each column. Ghobadloo (GHO), Girke (GIR), and Toghermezi (TOG). Chl a, Chl b, Chl a+b, CARs, S.O.V., and C.V. refer to chlorophyll a, chlorophyll b, chlorophyll a+b, carotenoids content, source of variation, and coefficient of variation, respectively.

**Table 3:** The ANOVA for the effects of PEG and sorbitol on the biochemical traits of three melon genotypes under in vitro conditions.

Mean Square							
S.O.V.	df	Proline	MDA	H_2_O_2_	TAA	TFC	TPC
Genotype	2	193.397 ^**^	65.835 ^**^	26.620 ^**^	14.484 ^**^	47.989 ^**^	829.397 ^**^
Drought	6	227.600 ^**^	27.153 ^**^	4.726 ^**^	10.926 ^**^	57.348 ^**^	763.995 ^**^
Genotype × Drought	12	126.294 ^**^	9.013 ^**^	0.266 ^**^	0.540 ^**^	6.179 ^**^	26.860 ^**^
Error	42	2.174	0.291	0.015	0.020	0.095	1.762
C.V. (%)		8.52	10.72	5.10	4.54	7.38	3.77

** indicated significance at 1% probability levels. S.O.V., df, and C.V. refer to the source of variation, degree of freedom, and coefficient of variation, respectively.

**Table 4:** The ANOVA for the effects of PEG and sorbitol on the total soluble protein content and the antioxidant enzyme activity of three melon genotypes under in vitro conditions.

Mean Square					
	df	TSP Content	APX Specialactivity	GPX Special Activity	SOD Special Activity
Genotype	2	2.212 ^**^	0.802 ^**^	4133.235 ^**^	2.256 ^**^
Drought	6	1.387 ^**^	0.627 ^**^	341.848 ^**^	0.584 ^**^
Genotype × Drought	12	0.146 ^**^	0.265 ^**^	62.531 ^**^	0.141 ^**^
Error	42	0.034	0.066	4.619	0.017
C.V. (%)		13.916	61.09	7.29	15.07

** indicated significance at 1% probability levels. S.O.V., df, and C.V. refer to the source of variation, degree of freedom, and coefficient of variation, respectively.

## Data Availability

The datasets generated and analyzed during the current study are available from the corresponding author upon reasonable request.
